# Reconciling Horse Welfare, Worker Safety, and Public Expectations: Horse Event Incident Management Systems in Australia

**DOI:** 10.3390/ani6030016

**Published:** 2016-02-24

**Authors:** Julie M. Fiedler, Paul D. McGreevy

**Affiliations:** 1 Faculty of Law, Education, Business and Arts, Charles Darwin University, Casuarina NT 0810, Australia; 2 Faculty of Veterinary Science, School of Life and Environmental Sciences, University of Sydney, Sydney NSW 2006, Australia; paul.mcgreevy@sydney.edu.au

**Keywords:** horse, risk, safety, injury, accident, management, mitigation, behaviour change

## Abstract

**Simple Summary:**

Although often highly rewarding, human-horse interactions can also be dangerous. Using examples from equine and other contexts, this article acknowledges the growing public awareness of animal welfare, work underway towards safer equestrian workplaces, and the potential for adapting large animal rescue skills for the purposes of horse event incident management. Additionally, we identity the need for further research into communication strategies that address animal welfare and safety issues that arise when humans and horses interact in the workplace.

**Abstract:**

Human-horse interactions have a rich tradition and can be highly rewarding, particularly within sport and recreation pursuits, but they can also be dangerous or even life-threatening. In parallel, sport and recreation pursuits involving animals, including horses, are facing an increased level of public scrutiny in relation to the use of animals for these purposes. However, the challenge lies with event organisers to reconcile the expectations of the public, the need to meet legal requirements to reduce or eliminate risks to paid and volunteer workers, and address horse welfare. In this article we explore incident management at horse events as an example of a situation where volunteers and horses can be placed at risk during a rescue. We introduce large animal rescue skills as a solution to improving worker safety and improving horse welfare outcomes. Whilst there are government and horse industry initiatives to improve safety and address animal welfare, there remains a pressing need to invest in a strong communication plan which will improve the safety of workplaces in which humans and horses interact.

## 1. Introduction

Sports that use animals can operate only with a social licence [1]. Public expectations surrounding the use of animals in sporting and recreational contexts are rapidly evolving and are being shaped, in part at least, by media stories of animals at risk, coupled with the use of graphic imagery. In parallel, there are strong public expectations of safe working environments for people, enforced by law, including those workplaces where there are human-horse interactions. The current article focuses on three areas in this domain: first, the increasing public awareness of animal welfare; second, the requirement for safer workplaces where both humans and horses are present; and third, the need for an industry-led communication plan that will address animal welfare issues and safer workplaces involving horses.

Horse event incident management provides a context to a feasible solution to all of these emergent issues. Large animal rescue skills provide a framework to manage a potentially hazardous equine patient-centred incident which, in turn, provides a safer workplace for people. The incident will be better managed if responders are trained and safe systems of work are adopted, leading to improved welfare outcomes for the horse.

In this article, animal welfare relates to the state of an animal in its attempts to cope with its environment [2] (p. 524). While our discussion is based on experience working with Australian horse-related sports and recreation activities, the issues discussed are relevant to other sport and recreation contexts involving animals, disparate organizations, and sub-groups, such as, cattle, sheep, and pigs used for exhibition or competition.

## 2. Public Awareness and Animal Welfare

Traditional newspapers, radio, and television, coupled with social media, offer an unprecedented opportunity to shape public consciousness on a wide range of issues. Social media platforms act as a carriageway for calls to action which, in turn, mobilise the online community to participate in targeted grassroots activism. One example of grassroots campaigning is the Australian platform “*Get Up!*” [3] and its project “*Community Run*” [4]. The GetUp! website claims that GetUp! has over one million members and lists a high court win among its achievements. Issues, including animal cruelty, are able to gain an increased profile through mediating technologies, particularly where graphic images boost a story’s impact.

In Australia, a recent newsfeed has featured animals-at-risk stories coupled with graphic images from the live cattle export trade following the ABC Four Corners program “*A Bloody Business*” [5] and the greyhound racing industry story “*Making a Killing*” [6]. Both documentaries have resulted in political and industry actions to improve animal welfare outcomes and manage threats to the long-term reputation of the organisations involved. Public outrage following exposure of animal cruelty in the live cattle export story resulted in actions which included the GetUp! 250,000 signature petition on live cattle export being presented to Prime Minister Julia Gillard [7], which contributed to the Australian Government suspending trade with Indonesia [8]. Similarly, public outrage following the broadcast of live-baiting practices in the greyound racing industry, has resulted in the removal of the Racing Queeensland board [9], the NSW Government establishing a Special Commissionof Inquiry [10], and the (custodial) sentencing of three greyhound trainers [1].

Citizens in many countries set expectations for animal welfare through elected government representatives and the making of laws. Examples of such law reform include the Swiss Government’s requirements for meeting the social needs of horses [11] and, more recently, the New Zealand government’s legal recognition of animal sentience [12]. In Australia, where animal law is not as advanced as in Europe or the UK, there is a boom in the tertiary study of animal law, perhaps due to the media profile given to animal welfare issues [13].

Modern technology provides easy access to information for individuals to become informed about animal welfare issues and provides the platform for animal welfare messages to be shared thousands of times, further contributing to the rapid shaping of public opinion. There are numerous examples of the media reporting animal welfare concerns and developments in public outrage and policy change. Indicative examples of such scenarios that have been reported in the media are outlined in Table 1.

## 3. Public Opinion and Horses at Risk

Media channels dedicated to horse-themed journalism provide a focus for the public’s increasing concern for animal welfare. An example is *Epona.TV’s* blog page [18] that, *inter alia*, publishes articles related to horses at risk of having their welfare compromised in sporting contexts. One blog example, “Akeem Foldager timeline” provides a chronicle of public, organizational, and industry participant actions following the publication of photographic evidence of a ridden horse being constricted by the two bits associated with a double bridle, resulting in the animal’s tongue turning blue [19]. The science underpinning cardiovascular changes that occur when restrictive gear is used in equitation is well established [20] and the principles of ethical equitation are reasonably clear [21]. However, there still remain considerable gaps between equine welfare science and mainstream horse use. To build an understanding of how attitude, beliefs and values differ between horse welfare advocates and professional horse industry participants, and how incremental improvements in horse welfare can be achieved, there is a need for further investment in research from the social science fields.

For the purposes of this article, a sport or recreation horse activity is defined as a structured, managed environment where the public is invited to view proceedings. In this discussion, we focus on the type of competitions conducted under the rules of an incorporated association; for example, dressage, horse-racing, and endurance trials. Such competitive events attract competitors and spectators, with the latter including online or offline support crews, organisational members, officials, and the general public. Throughout the events, participants may use social media to share opinions in real-time and worldwide. As an example, if a horse’s safety appears to have been compromised, opinions are formed and shared by the public without consent from the event organisers, horse owner, or competitor. The online dialogue may attract particular interest and following if the participants are injured, a horse is trapped, or a poor response is mounted by officials. Some examples of such scenarios are outlined in Table 2.

The shaping of public opinion has broadened to include the management of deceased animals, with a 2014 billboard featuring a deceased racehorse erected in Melbourne prior to the spring racing carnival [25], and the 2015 billboards on busses [26], displaying an image of a deceased cow, broadcasting a message against live export. The growing public scrutiny surrounding the euthanasia of horses is providing a rapidly evolving communication challenge for event organisers and veterinarians, charged with ensuring the welfare of participating horses is paramount. Traditional values, practices, and policies may be well accepted by participants but often do not have the same meaning for the general public. For community-level events, the handling of deceased horses with dignity is introduced in the Australian Horse Welfare and Well-being Toolkit [27] (p. 25). Covering planning, logistics, veterinary support and data collection, the toolkit sets out a checklist for organisers. However, there is no independent online portal of evidence-based information for the public and journalists seeking information on the welfare of sport and recreation horses, including those times when euthanasia is recommended.

## 4. Government Efforts to Manage Risks Posed by Horses in Workplace

Workplace safety laws, workers compensation claims and fines following the death of Sarah Waugh [28] are driving government-led initiatives targeting safety in workplaces involving horses. *Worksafe Australia* statistics reveals that, on average, one horse industry worker is hospitalised per day in Australia from a fall, kick, strike, or bite [29] (p. 2).

Workplaces involving horses have inherent risks for workers [30] (p. 324). Under Australian law, any horse organisation that engages a paid staff member or contractor is considered a “Person or Organisation Conducting a Business Undertaking (PCBU)” and Workplace Health and Safety laws apply. In Australia, competitive horse events, other than racing, are almost exclusively organised and managed by a volunteer workforce under the auspices of a peak body that employs staff in a national, regional, or state office. Therefore, the PCBU status applies to many horse events and the requirement for volunteer workplace inductions, defined job roles, and the use of personal protective equipment (PPE) applies.

Australian government agencies have taken action to manage some horse-related risks. These include development Worksafe Australia’s “*Guidelines for reducing risk when new and inexperienced people interact with horses*” [29], the Australian Government Australian Skills and Quality Authority’s report “*Training in equine programs in Australia*” [31] and implementation of Technical and Further Education TAFE New South Wales “*Procedures for delivery of horse industry training*” [32]. Media stories providing examples of government efforts to manage risks appear in Table 3.

The level of risk increases for emergency service workers when attending an incident scene involving a horse. In research undertaken by Smith *et al.* [35] (p. 9), fire and rescue volunteers reported a concern about the physical management of large animals, inter-agency coordination, and dealing with owners to the extent that they seek further training opportunities in this domain. It is acknowledged that large animal rescues expose humans to a range of risks, including an unpredictable working environment attributed to horse behavioural characteristics, biosecurity, heavy manual handling, and injuries from being kicked.

Actions to manage risks undertaken by the South Australian and New South Wales State Emergency Services have resulted in the preparation of large animal rescue technical manuals, delivery of standardised training and purchase of fit-for-purpose equipment. The capabilities of the emergency response agencies are enhanced through partnerships with organisations specialising in emergency animal patient care, including Equine Veterinarians Australia’s Large Animal Rescue Registry [36], RSPCA state bodies, and the non-profit South Australian Veterinary Emergency Management [37].

To date, there has been only limited investment by government or the horse industry in safety research or data collection systems that have the potential to result in safer human-horse interactions in the workplace.

## 5. Horse Industry Efforts to Manage Risks

In 2014, the Australian Horse Industry Council (AHIC) conducted a national survey of horse owners and industry workers [38]. Of the 3017 responses analysed, 38% of respondents had received a horse-related injury serious enough to require hospitalisation. After horse-racing, sports including polo, polocrosse, and events involving cattle (e.g., camp drafting), recorded the highest reported incidences of personal injury during training and competition. Furthermore, from the 2083 responses analysed, eight out of ten people at horse events are volunteers, with 31% volunteering at least monthly. The number of participants in the Australian horse industry is not known, so an incidence rate cannot be calculated.

The AHIC has addressed risks to horses and humans through the *HorseSafe Code of Practice* [39]. The voluntary *Code* provides a minimum standard for assessment and control of risks associated with people working around horses while horse welfare is the focus of the *Australian Horse Welfare Protocol* [40].

Horse industry participants have been further supported by evidence-based tools developed by the *International Society for Equitation Science* (ISES), including the ISES *Code of Conduct* that offers guidelines to ensure optimal horse and rider welfare and safety at competitive events. This code refers to other ISES position statements and embraces the *ISES’s First Principles of Horse Training* [41] which, in turn, informs the selection and application of handling techniques.

Targeting horse event organisers, the Australian Horse Industry Council has developed the *Australian Horse Welfare and Well-being Toolkit* [27]. This resource provides an introduction to horse event incident management and recommends the appointment of a Horse Welfare Officer. The role of the officer is to work across organisational management structures, with oversight of the welfare of horses, including when an incident occurs [27].

## 6. Horse Event Incident Management

In this article, we have so far acknowledged the increased public awareness of animal welfare issues, the risks in workplaces where humans and horses interact, and government and horse industry efforts to promote safer work practices. We now showcase one solution for providing a safer working environment when humans and horses are involved in an event incident.

Technical rescue knowledge and skills drawn from the emergency services sector provide a potential model for adaptation to horse incident management. In particular, there are procedures that emerge from the emergency services sector. This includes preparation of an incident management plan, use of established communication systems, and selection of trained personnel for a safer work environment [42] (p. 15). Similar to human-centred incident responses, an assessment may determine that an equine trauma care patient can be transferred to a place of safety, triaged away from public scrutiny and, on occasion, prepared for transit to an equine hospital [43] (p. 80).

**Figure 1:**
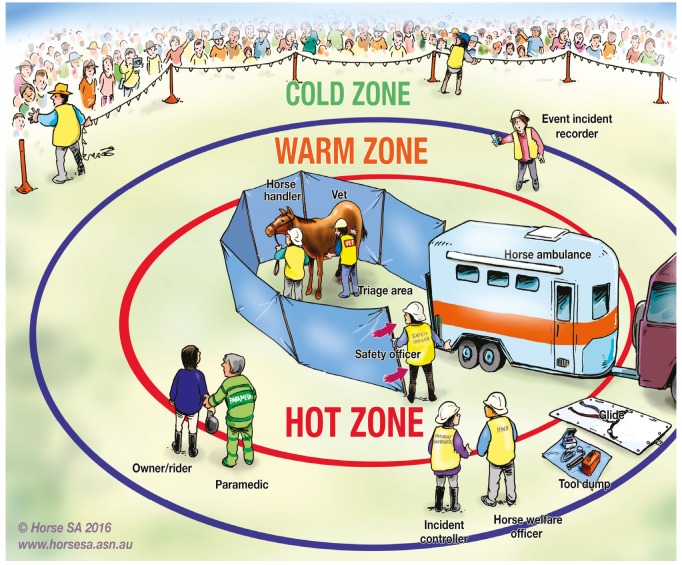
Large animal rescue three working zones.

Large animal rescue (LAR) adopts an even-handed approach, balancing high-risk hazard management with the welfare of the horse. To avoid further injury to humans or horses, the management of an incident scene requires the assignment of roles to individual responders, the use of personal protective equipment and the establishment of three working zones (Figure 1), based on the level of risk [44] (pp. 6–10). The first, a “hot zone” nearest to the equine patient, has the highest risk, where only essential personnel are positioned. The second, a “warm zone”, is outside of the kicking or head-tossing range of the horse, where the incident controller, safety officer, veterinarian, horse welfare officer, owner, and tool dump are positioned. The third is the “cold zone”, a low-risk area where the media and spectators are positioned [27] (p. 24). In most situations, An introductory level LAR kit consists of strops (or straps), pole hooks to use as extensions of responders arms, a tool to thread the strops under the horse, rope, and a rescue glide [44] (pp. 111–113).

**Figure 2:**
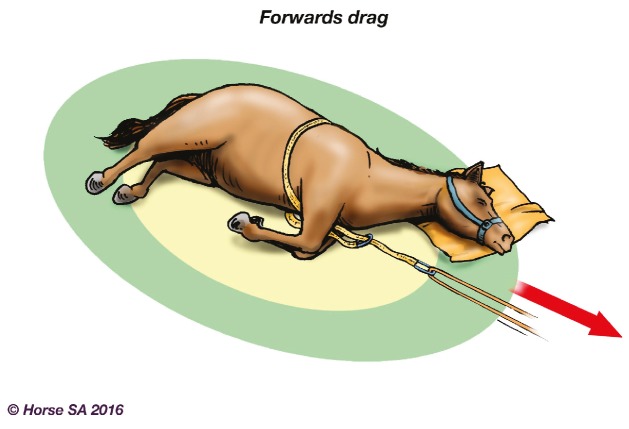
Forwards drag.

**Figure 3:**
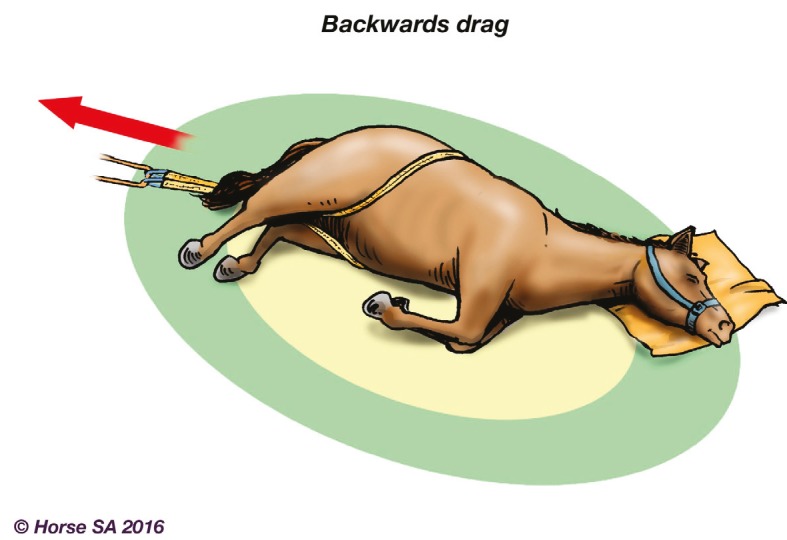
Backwards drag.

**Figure 4:**
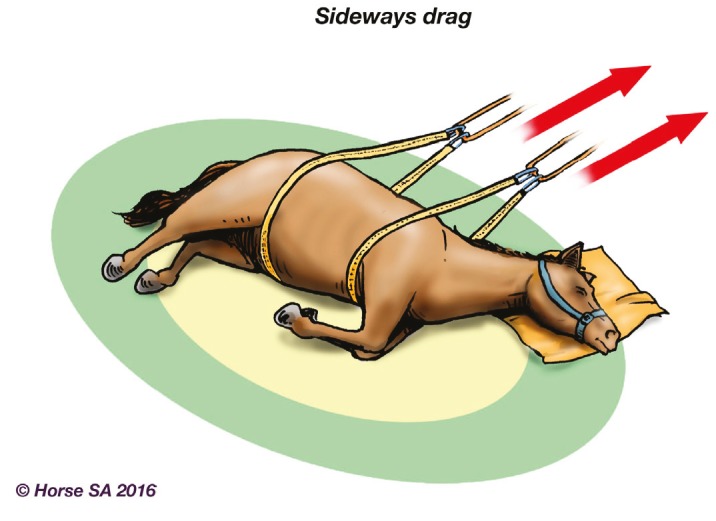
Sideways drag.

**Figure 5:**
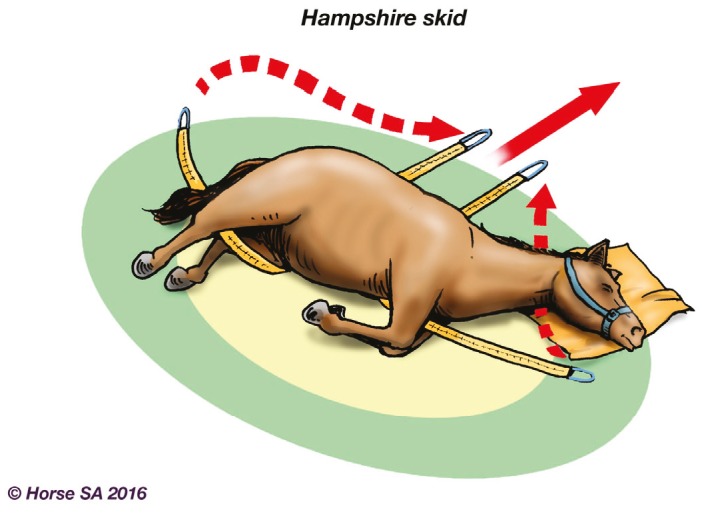
Hampshire Skid.

**Figure 6:**
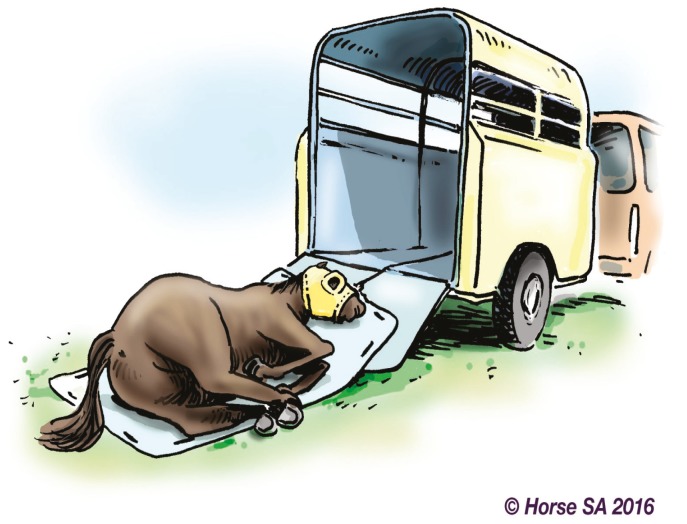
Horse on a rescue glide.

Basic LAR manipulation techniques, aimed at manoeuvring the horse to a safer place, include the forward drag (Figure 2), backward drag (Figure 3), sideways drag (Figure 4) and the so-called Hampshire skid (Figure 5) [45] (pp. 21–25). The torso of the horse is used for manoeuvring, as use of the head, neck, legs or tail may result in further injury [44] (pp. 37–38). With a recumbent horse, the dependent eye needs to be protected [42] (p. 277). The techniques may also be used to place a live or deceased horse onto a rescue glide (Figure 6) which, in turn, is winched into the float [44] (p. 116), and transferred to a place of safety.

In these scenarios, a veterinarian works as a part of the horse event incident response crew in the same way a paramedic integrates into a human rescue scenario. Large animal rescue provides a casualty-centred approach, incorporating triage, immediate care and, if required, euthanasia, while all the time keeping people safer in what is often a dynamic rescue environment. These characteristics of a horse-related emergency mean that optimal handling of such incidents merits careful planning lest it falls under the glare of intense public scrutiny [46]. Horses that have had their innate sense of safety compromised, such as when an incident occurs, may react unpredictably and cause severe injury to themselves or people. Planned and managed incident responses provide an opportunity to improve horse welfare outcomes through the benchmarking of key outcomes, including response time, survival rate of equine patients and, for human responders, injury statistics. Through the provision of rescue and trauma care training, risks relating to human safety and horse welfare are mitigated [43] (p. 80).

Although many participants in horse incident management are volunteers, it should be noted that the Australian workplace safety laws requiring PCBUs to minimize or eliminate risk in the workplace [47] (p. 1) may still apply. Therefore, horse events, if categorized as a PCBU, have a legal requirement for a safe workplace. At events, volunteer workers may lack experience with horses, but nevertheless supervise subordinates and others [29] (p. 19). Volunteers may also be expected to deal with horse-related incidents, in addition to their regular role at the event, even though they may not have been provided with specialist training. This approach aligns with the observations of Thompson *et al.* [48] (p. 565), who note that horse riders and handlers often undertake activities a certain way simply “because they have always been done that way”, and are rarely provided with advice on how to reduce or remove risks. A risk assessment by event organisers may determine that little capacity exists amongst the volunteers to respond effectively to an incident and, therefore, pre-event planning will need to address this gap.

Public scruity of horses involved in incidents will attract media attention, as will the manner in which any response is undertaken. For examples of media stories refer to Table 4.

## 7. Communication Strategy Design

Our discussion leads us to propose the need for an industry-led communication strategy that addresses the dual messages about the growing public awareness of animal welfare issues and safer workplaces where interactions with horses take place. A communication plan should set a range of measurable targets, including the training of horse event volunteers in large animal rescue skills and educating the public on expectations relating to horse welfare in the settings of sport and recreation.

Few education or training resources are available to support horse event organising committees or volunteers. For emergency service agencies and horse owners, educational resources set in the context of the natural environment, farms, road transport and equestrian enterprises or workplaces include the books *Technical Large Animal Emergency Rescue* [42] and *Equine Emergency Rescue* [44].

However, there is a gap in resources to support volunteers responsible for managing horse event incidents where the additional elements of crowding by spectators and public scrutiny of animal welfare outcomes are factors contributing to a successful resolution. Horse event volunteers are unlikely to be experienced in working safely as part of an incident management team or be proficient at handling horses in stressful situations.

Forming a core element of a communication strategy, the development of targeted educational resources can provide guidance for safer practices in the workplace, and influence the adoption of new techniques. To ensure relevance for event volunteers, resources and training should be culturally appropriate, customised to address recognisable incidents, and easily accessible for “just in time” reminders. Communication prepared by organisers prior to the event, targeting participants and spectators, may help to manage expectations of incident responses and horse welfare outcomes.

Barriers to an effective change in practices may include horse industry participants’ attitudes to injury, with many participants currently accepting injury as part of the job [50] (p. 897). Similarly, Thompson *et al*. [48] observes that throughout history, horses and riding have been described as forms of “art” that conflict with the practical application of risk mitigation. These authors go on to argue that the need for the horse to be safe could be reconfigured in a way to keep riders safe, too.

We hypothesize that if horses and people are kept safer in the workplace, public expectations relating to animal welfare are more likely to be met. An example of using the needs of an animal to promote safer decision-making can be drawn from the disaster management sector, notably Australian bushfire evacuation planning [51]. The Australian *National Planning Principles for Animals in Disasters* states that human welfare and safety will be improved if emergency management planning processes include animals [52]. Furthermore, the presence of animals in emergency situations impact human behaviour and safety; therefore, emergency service organisations need to work with communities on animal emergency management above standard preparedness (p. 7).

The Royal Commission into the 2009 Victorian Bushfires found that people died as they chose to stay with their pets or, because of their pets, they left too late [53] (p. ii). Through recognising the human-animal relationship [54], public messaging can encourage owners to take their pets to a safer place, which increases the liklihood that the humans will also stay safe. When the LAR ethos is applied to a horse event incidents, horse-centred messaging results in caring for the horse as a casualty that, in turn, creates a safer workplace [43] (pp. 77–81).

Development and implementation of an industry-led communication plan is likely to be more effective than if initiated by a non-sectorial industry body, for example, the Australian Horse Industry Council [55], through a participatory model that involves horse sport and recreation participants, veterinarians, and large animal rescue experts. We acknowledge that communication strategies will need to be designed to suit different horse sport and recreation activities, recognising the different drivers for participation and that the contexts in which horses perform have a wide range of variability.

## 8. Conclusions

In this article, we have discussed the increased public awareness of animal welfare issues, the risks in workplaces where humans and horses interact and government and horse industry efforts to promote safer work practices. We have emphasised the need for a communication plan that addresses the paired messages of growing public awareness of animal welfare issues and safer workplaces where humans and horses interact. We recommend further research into factors that will reduce risks in the workplace involving human-horse interactions. Furthermore, we recommend development of an industry-led communication plan which is undertaken in partnership with animal welfare advocacy organisations and experts from the fields of media communication and social sciences. The plan would set out measurable targets, including the training of horse event volunteers in large animal rescue skills and educating the public on expectations relating to horse welfare in the settings of sport and recreation. The plan will need to clearly identify how it will be implemented, maintained, and undergo evaluation by an independent organisation. A well-designed communication plan will, in turn, lead to a safer workplace for people interacting with horses.

## Figures and Tables

**Table 1:** Examples of public awareness and animal welfare media stories.

Media	Animal Welfare Aspect	Storyline
It was Spain’s “national fiesta”. Now bullfighting divides its people [14]	Bulls are killed for public sport and entertainment	Next generation of youth does not see a role for bullfighting in Spain in an increasingly globalized world
Zimbabwe bans lion hunting after international outcry [15]	Lions, tigers and other exotic wildlife hunted for sport, offered as packaged tourism experiences	An international public outcry arising from the killing of a favourite lion by a U.S. citizen results in changes to hunting laws
Pigs to use Twitter and Facebook to challenge animal welfare criticism [16]	The housing conditions and care of animals raised for human consumption	Farmers use social media to inform public on how farm animals are raised
Starbucks to switch to 100 percent cage-free eggs by 2020 [17]	Ethical choices when sourcing ingredients	A commercial decision by Starbucks to remain competitive

**Table 2:** Examples of public awareness and horses at risk media stories.

Media	Animal Welfare Issue	Storyline
Pictures of lame horse a PR disaster on the magic day Black Caviar came back to Sydney [22]	A lame horse was ridden by a reporter during the broadcast of an interview with the jockey of the winning horse, *Black Caviar*	The commercial decision by Channel 7 to continue with broadcasting the interview. The television station accused of a lapse in a duty of care
Clydesdale slips on Granite Island causeway [23]	Horse slips over on wet rubber matting, remaining recumbent for a period	Spectator reports the incident to RSPCA
Swiss Federation bans use of draw reins in 2016 [24]	Horses ridden with hyper flexed necks are under stress	Swiss Equestrian Federation moves to act on negative social media and calls for support of improved horse welfare by all equestrians

**Table 3:** Examples of media stories of government efforts to manage risks.

Story	Safety Issue	Storyline
State to regulate equine industry after death of Hunter rider Sarah Waugh [28]	Injuries and death to people who work with horses	Public awareness raised by parents of Sarah Waugh, resulting in development of an enforceable Code of Practice by Workcover NSW
Three Queensland vets face prosecution over how they managed Hendra cases [33]	Laws require that workers need to take reasonable care of themselves and others in the workplace	Regulators act to enforce Workplace Health and Safety laws
Improving safety in horse racing: it’s all in the data [34]	Research on the costs of Workers Compensation for racing industry riders	Development of a tool for comparing costs and risks associated with introducing strategies to improve safety

**Table 4:** Examples of media stories relating to horse incidents.

Story	Management	Storyline
Shocking picture shows racehorse champion *Wigmore Hall* destroyed at packed course [49]	Horse racing incident response	Incomplete screening of a horse racing incident resulted in the witnessing of the euthanasia of *Wigmore Hall*. The photograph was subsequently published on the front page of the *Daily Mirror*
Why the long face motorists? Farce as M6 is shut in both directions after horse gets stuck in its horsebox [7]	Emergency management procedures followed for a patient-centred rescue	Traffic delays as a trapped horse was extricated from a horse box on a busy motorway
